# HRP-OG: Online Learning with Generative Feature Replay for Hypertension Risk Prediction in a Nonstationary Environment

**DOI:** 10.3390/s24155033

**Published:** 2024-08-03

**Authors:** Shaofu Lin, Haokang Yan, Shiwei Zhou, Ziqian Qiao, Jianhui Chen

**Affiliations:** 1 Faculty of Information Technology, Beijing University of Technology, Beijing 100124, China; linshaofu@bjut.edu.cn (S.L.); zamaochick@163.com (S.Z.); 1766485082@emails.bjut.edu.cn (Z.Q.); 2 Beijing International Collaboration Base on Brain Informatics and Wisdom Services, Beijing University of Technology, Beijing 100124, China; 3 Beijing Key Laboratory of MRI and Brain Informatics, Beijing University of Technology, Beijing 100124, China; 4 Engineering Research Center of Intelligent Perception and Autonomous Control, Beijing 100124, China; 5 Engineering Research Center of Digital Community, Ministry of Education, Beijing 100124, China

**Keywords:** hypertension risk prediction, reinforcement learning, generative replay, online learning, electronic health records

## Abstract

Hypertension is a major risk factor for many serious diseases. With the aging population and lifestyle changes, the incidence of hypertension continues to rise, imposing a significant medical cost burden on patients and severely affecting their quality of life. Early intervention can greatly reduce the prevalence of hypertension. Research on hypertension early warning models based on electronic health records (EHRs) is an important and effective method for achieving early hypertension warning. However, limited by the scarcity and imbalance of multivisit records, and the nonstationary characteristics of hypertension features, it is difficult to predict the probability of hypertension prevalence in a patient effectively. Therefore, this study proposes an online hypertension monitoring model (HRP-OG) based on reinforcement learning and generative feature replay. It transforms the hypertension prediction problem into a sequential decision problem, achieving risk prediction of hypertension for patients using multivisit records. Sensors embedded in medical devices and wearables continuously capture real-time physiological data such as blood pressure, heart rate, and activity levels, which are integrated into the EHR. The fit between the samples generated by the generator and the real visit data is evaluated using maximum likelihood estimation, which can reduce the adversarial discrepancy between the feature space of hypertension and incoming incremental data, and the model is updated online based on real-time data using generative feature replay. The incorporation of sensor data ensures that the model adapts dynamically to changes in the condition of patients, facilitating timely interventions. In this study, the publicly available MIMIC-III data are used for validation, and the experimental results demonstrate that compared to existing advanced methods, HRP-OG can effectively improve the accuracy of hypertension risk prediction for few-shot multivisit record in nonstationary environments.

## 1. Introduction

Hypertension is a leading cause of early demise and debility globally [1]. It is responsible for around 7.5 million deaths per year, accounting for 12.8% of all deaths worldwide and about 18% of global deaths from cardiovascular disease [2]. Timely detection of the preclinical or high-risk stage of hypertension, namely, hypertension risk prediction, is very important. Clinical studies have shown that for individuals in the preclinical or at-risk stage, significant reductions in the progression of hypertension can be achieved through lifestyle changes or effective drug treatments [3]. However, due to limited medical resources, relying solely on healthcare workers to achieve hypertension risk prediction is not an easy task. Related research shows that about 1.13 billion people in the world are troubled by elevated blood pressure, but only about 50% are aware that they are hypertensive [4]. Hypertension is a gradual process, and specific symptoms may manifest before the onset of the disease. In this case, by analyzing EHRs, medical AI is able to understand the physical condition of patients to determine their future risk of developing hypertension [5]. Therefore, hypertension risk prediction assisted by artificial intelligence (AI) has become an urgent need and is one of the current research hotspots in medical AI.

Electronic health records (EHRs) are digital records that contain complete personal and health information of patients along with their medical history [6]. With the rapid development of EHRs, various medical AI applications based on EHRs have received widespread attention, including disease risk prediction systems [7,8,9], clinical knowledge question answering systems [10,11,12], drug recommendation systems [13,14], etc. Chronic disease risk prediction based on EHRs is an important way to achieve AI-assisted chronic disease warning, including hypertension risk prediction. Existing studies focus on developing complex models [15,16], especially deep neural networks, to extract feature information from multivisit EHR data for accurately predicting chronic disease risk. They depend on large-scale and high-quality multivisit EHRs.

**Figure 1:**
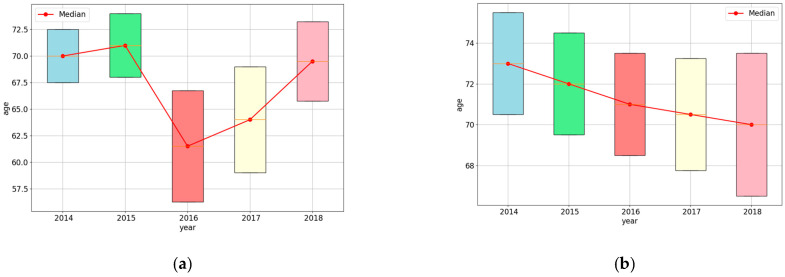
Distribution of age over 5 years. (**a**) Distribution of age in hospital 1; (**b**) distribution of age in hospital 2.

However, although hospitals worldwide have established sophisticated hospital in-formation systems, there are still differences in the storage methods and the data quality of EHRs between different hospitals [17]. At the same time, there are also challenges such as data ownership and privacy protection [18]. Therefore, it is difficult to integrate long-term data from multiple hospitals at once, to build a large-scale and high-quality multivisit EHRs dataset. In practical settings, chronic disease risk prediction modeling based on EHRs is often conducted in a few-shot and nonstationary environment. Few-shot EHR data are gradually generated and collected in batches. There are also significant differences in the distribution of data attributes for each batch. Figure 1 shows the statistics of EHR data of hypertensive patients from two hospitals in Hainan Province, China, demonstrating the distribution of the age of patients examined in each of the 5 years. It can be seen that the age characteristics of patients continuously change over five years. In subplot a of Figure 1, the age of patients ranged from 67.5 to 72.5 in 2014, increased in 2015, and then dropped significantly to 56–67 in 2016, before gradually rising again to 66–73. In subplot b of Figure 1, the age of patients consistently decreased from 70.6–75.8 to 66.5–73. In both datasets, the age distribution of patients is different in each year, and such changes lead to a fixed hypertension prediction model being unable to meet the actual needs of the patients.

Such a few-shot and nonstationary environment leads to adversarial differences between the feature space of disease prediction models and the continuously inputted incremental EHR data, which brings about the need for online learning of the model. However, the factors affecting hypertension risk are often multifaceted, requiring high-dimensional data for prediction [19]. Existing online learning methods struggle with learning high-dimensional data features. For examples, Ning et al. [20] proposed a robust algorithm using polar decomposition for derivation, transforming the learning problems into optimal feedback control problems for a group of low-dimensional linear systems and using optimal control to achieve learning from high-dimensional online data. However, such complex deep learning models tend to overfit to noisy data when the data are small [21]. When data distribution changes significantly, excessive adaptation to new data will also lead to a decrease in the generalization ability of the model.

Based on the above observations, this paper proposes a new hypertension risk prediction model, called HRP-OG, which combines reinforcement learning and generative feature replay techniques to predict hypertension risk based on multivisit EHR data. The model can be updated with incoming incremental data in a few-shot and nonstationary environment, achieving more accurate prediction of hypertension risk. The main contributions are as follows:

1. This paper transforms the hypertension risk prediction problem into a Markov decision process problem and proposes a hypertension monitoring model based on reinforcement learning. A new dual experience replay strategy is proposed to further distinguish the high and low risk experiences for reducing the bias caused by the imbalance distribution of multivisit EHRs and improving the few-shot learning capability of the model.
2. A new generator updating strategy is proposed. By comparing the fitting degree between the incremental data and historical data with the hypertension feature space, the generator adopts different updating modes for reducing the adversarial differences between the incremental EHR data and the feature space.
3. This paper constructs a 12-year continuous hypertension risk prediction dataset from the public MIMIC-III dataset to simulate a few-shot, incremental, and nonstationary EHR generation scenario. Experiments are conducted on this dataset and the results demonstrate that the proposed HRP-OG can provide effective and continuously updating hypertension risk prediction in a few-shot and nonstationary environment.

The rest of this paper is organized as follows. Section 2 reviews machine learning methods for chronic disease prediction. Section 3 introduces the proposed HRP-OG model. Section 4 describes the datasets and compares the performance of this model with the baselines. In Section 5, the performance of this model is compared and analyzed with the variants. Finally, Section 6 gives the conclusions and future work.

## 2. Related Work

Hypertension risk prediction is very important and effective for reducing the prevalence of hypertension. Traditional hypertension risk prediction is mostly achieved using regression models [22]. These methods depend on high-quality medical resources and have difficulty meeting the needs of a large population at high risk of hypertension. Medical AI provides an effective way to alleviate the pressure on medical resources for chronic disease (e.g., hypertension) risk prediction. Its typical application is EHR-based chronic disease risk prediction.

EHR-based chronic disease risk prediction refers to predicting future disease risk by analyzing previously observed EHR information [23,24]. Early researchers focused only on the current health status of patients and tended to model single-visit EHR data for predicting chronic disease risks. For example, Syed et al. [25] integrated neural network and genetic algorithm to predict the probability of heart disease based on 12 factors, including family history, diabetes, hypertension, smoking status, obesity, etc. They used the genetic algorithm for a global search to optimize network initialization weights. Sayali et al. [26] combined Bayesian and K-nearest neighbor (KNN) algorithms to predict whether a patient has heart disease based on symptoms. Based on this, they proposed a Convolutional Neural Networks-based Uni-Modal Disease Risk Prediction (CNN-UDRP) algorithm that uses a convolutional neural network to further predict the disease risk of the patient.

However, the development of chronic diseases is a long-term process, and it is difficult to obtain more accurate prediction results by only focusing on the current health status of patients. Therefore, more and more researchers no longer focus solely on the horizontal features of EHRs but begin to combine the current and historical EHRs of patients to extract longitudinal EHR features [27,28]. Multivisit EHRs are used to predict chronic disease risks. For example, Gao et al. [29] used a phase-aware neural network to extract disease stage information from the time series of EHR data for health risk prediction. Zhao et al. [30] developed a model to extract and utilize the uncertainty between multiple visit intervals of EHRs for improving disease prediction. Nancy et al. [31] integrated recursive neural network and bidirectional long short-term memory (Bi-LSTM) to manage continuous EHR time series and build a model for predicting heart disease risks. Most of these studies adopted complex deep learning models, which depended on large-scale and high-quality multivisit EHR data.

In practical settings, it is difficult to obtain large-scale and high-quality multivisit EHR data at once. Therefore, online learning of models is gradually gaining attention for EHR-based chronic disease risk prediction. Online learning is a particular incremental learning method [32] that refers to the paradigm where a model is updated iteratively and adaptively over time as new data become available in a sequential manner [33]. For chronic disease risk prediction, the main methods of online learning can be broadly classified into three categories: regularization-based methods, dynamic network structure-based methods, and replay-based methods [34]. Regularization-based methods [35] mitigate catastrophic forgetting by imposing constraints to enable the updating of network parameters. However, because of penalizing changes to important parameters, the regularization term may lead to poor adaptability of the model, making it difficult to adapt to new tasks with different data distributions and features. Dynamic network structure-based methods [36] dynamically adapt the network to new tasks. However, due to the continuous complexity of the model structure, this kind of method is unstable during the model update process and is prone to overfitting on few-shot data. Therefore, these two kinds of online learning methods are not suitable for hypertension risk prediction in a few-shot and nonstationary environment.

Replay-based methods rely on memory replay, meaning that samples from previous tasks are replayed when the samples from the current task are used for learning [37]. It can effectively retain historical samples while not requiring adjustments to the model structure. Therefore, it does not have the aforementioned drawbacks and is currently a research hotspot in online learning for chronic disease prediction models. For example, Wang et al. [38] proposed a novel method named embedding episodic memory and consolidation to prevent catastrophic forgetting on disease diagnosis tasks. This method retrieved a replay batch from the memory set and leveraged medical domain knowledge, incorporating both context and medical entity features to transfer knowledge to new stages. Gao et al. [39] proposed a brain disease prediction method that combines a multiloop learning algorithm with a generative adversarial network. The method combines evidence with better sample contribution ranking during training processes, selecting 1/2 of the samples each time for learning and generating samples to input into the database. In chronic disease risk prediction, EHR data are characterized by continuous changes and interconnections. Combining historical data with current data can achieve better predictions, making the replay-based methods highly suitable.

However, when there is a significant difference in the feature distribution between new data and historical data, the accuracy of replay-based methods will be poor. The physiological characteristics of high-risk individuals for hypertension are influenced by various factors such as climate and psychology. Therefore, there are significant differences in the distribution of attributes between different batches of data in few-shot and nonstationary environments. In this case, using existing replay-based methods for online learning of the model may cause differences between the generated samples and the feature space of hypertension, thereby affecting the accuracy of the model. Therefore, it is necessary to innovate existing replay-based methods to achieve online learning of the hypertension risk prediction model in the few-shot and nonstationary environment.

## 3. Methods

### 3.1. The Model Framework of HRP-OG

**Figure 2:**
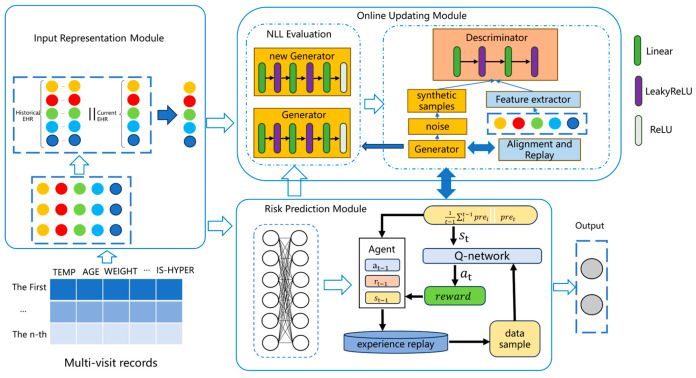
The whole framework of HRP-OG.

This study proposes an online hypertension risk monitoring model based on reinforcement learning and generative feature replay. The overall framework consists of three parts: the input representation module, the hypertension risk prediction module, and the model online updating module. The specific model framework is illustrated in Figure 2.

### 3.2. The Input Representation Module

The input representation module converts each visit record into a multidimensional input embedding that serves as the input to subsequent modules. In EHR data, each visit record is represented as $P^{n}=\{A^{n},{IS-HYPER}^{n}\}$, where n represents the n-th patient and IS-HYPER represents the sample tag. A represents the input embedding of record containing the above 20 attributes, which can be expressed as follows:

(1) $$A={\left|\right|}_{i}^{\left|M\right|}item\left[i\right]$$

where item[i] is the input embedding corresponding to the i-th attribute of the visit record, |M| represents the number of attributes, and || represents the concatenation of $item\left[1\right]$, $item\left[2\right]$ … $item\left[\left|M\right|\right]$.

### 3.3. The Risk Prediction Module

The hypertension risk prediction module pools all attribute embedding sequences transformed from longitudinal EHR data, which are based on the reinforcement learning framework, to perform the hypertension risk warning task and record the changes in prediction states for patients. In addition, the unique reward, penalty functions, and sample selection strategies can further improve prediction accuracy.

This study abstracts the hypertension risk prediction module as an agent, in which the visit record of each patient serves as a state and all EHR data as the environment. The choice of predicting hypertension risk for patients is considered as an action, and the accuracy of hypertension risk prediction is the reward. Ultimately, the agent provides hypertension risk predictions based on the current environment.

At the beginning, we set the state of the patient as 0 vector. At time t, we update the state vector of the patient by connecting the historical visit records with the current visit record of the patient; the state can be calculated as follows:

(2) $${state}_{t}^{n}=\sum_{i=0}^{\left|M\right|}{item\left[i\right]}_{i}^{n}||\frac{1}{t-1}\sum_{i}^{t-1}P_{i}^{n}$$

The agent will predict the hypertension risk of the patient. The actions of the agent are divided into two categories, which can be expressed as follows:

(3) $$a_{t}=\left\{\begin{array}{c} 1\;\;\;is\_hyper\\ 0\;\;\;no\_hyper \end{array}\right.$$

After the agent performs the action, the environment evaluates whether the hypertension risk prediction for the patient is correct and provides a reward signal to the agent. The reward function consists of two parts, which can be expressed as follows:

(4) reward=reward1+reward2

where reward1 represents the judgement of whether the hypertension risk prediction is correct, and it can be calculated as follows:

(5) $$reward1=\left\{\begin{array}{c} -1\;if\;a!=lable\\ 1\;if\;a==lable \end{array}\right.$$

where label represents the sample tag of visit record. reward2 represents the reciprocal of the distance between the predicted and true results, and it can be calculated as follows:

(6) $$reward2=Sigmoid\left(\frac{1}{\sqrt[{2}]{{\left(left-is\_hyper\right)}^{2}-{\left(right-no\_hyper\right)}^{2}}}\right)$$

where left and right values are obtained through the DQN network and represent the probabilities of the patient having hypertension and not having hypertension, respectively. The agent predicts the risk of hypertension by comparing these probabilities and selecting the larger one.

This study uses a DQN network to receive the current visit data status of the patient transmitted by the agent. The DQN stores the current health status of the patient, predicted disease condition, reward value, and the last health status in a dual replay buffer. The dual replay buffer stores data in the corresponding buffer based on whether the current versus the last visit has changed. The main network is trained by sampling data samples from the buffer according to the sampling strategy, aiming to fit the real hypertension condition as closely as possible after training. Finally, the loss function is calculated based on the Q-values of the main network and the target network, and the network parameters are updated using historical data.

To expedite the model training process, we design a time-based priority allocation strategy and a priority-based sampling strategy. In the allocation strategy, the DQN network calculates the difference between the estimated value of the action and the current function output value. The priority increases as the error grows larger. The gap value can be calculated as follows:

(7) $$gap\left(s_{t},a_{t},r_{t},s_{t+1}\right)=r_{t}+y\begin{array}{c} max\\ a_{t} \end{array}Q\left(s_{t+1},a_{t+1}\right)-Q(s_{t},a_{t})$$

where y represents the discount factor, $y\begin{array}{c} max\\ a_{t} \end{array}Q\left(s_{t+1},a_{t+1}\right)$ represents the target value, and $Q(s_{t},a_{t})$ represents the current value.

After calculating the priority scores of the samples, the model uses the softmax function to map the gap values, converting the priority scores of the data samples into probabilities of being selected from the buffer. The value can be calculated as follows:

(8) $$P(n)=\frac{e^{{gap}^{n}}}{\sum_{i=1}^{n}e^{{gap}^{i}}}$$

where P(n) represents the probability of the n-th data sample being selected from the buffer. After assigning sampling probabilities to each sample in the buffer, the DQN network selects a batch of data samples for learning based on these probabilities. To better extract sample data, we propose a dual experience replay strategy, establishing two buffers to store the samples predicted correctly and incorrectly. We define the loss function as follows:

(9) $$L=\varphi_{1}\sum_{t\in P_{a==lable}}{gap\left(s_{t},a_{t},r_{t},s_{t+1}\right)}^{2}+\varphi_{2}\sum_{t\in P_{a!=lable}}{gap\left(s_{t},a_{t},r_{t},s_{t+1}\right)}^{2}$$

where $\varphi_{1}$ and $\varphi_{2}$ represent the weights used to select data samples from the double experience buffer. When selecting data samples from the first buffer, we set $\varphi_{1}=1$ and $\varphi_{2}=0$. Conversely, when selecting data samples from the second buffer, we set $\varphi_{1}=0$ and $\varphi_{2}=1$. $t\in P_{a==lable}$ represents the buffer where data samples have the same action values and label values, while $t\in P_{a!=lable}$ represents the buffer where data samples have different action values and label values.

### 3.4. The Model Updating Module

The model online updating module uses maximum likelihood estimation to evaluate the distance between old data features and new data features. By utilizing a generator, discriminator, and replay alignment strategy, the generalization ability of the model is enhanced.

When the t-th batch of data arrives, the model does not directly feed the data into the hypertension risk warning module for learning. Instead, it generates a new generator $G_{t}$ to learn the feature distribution of the new data. At the same time, it calls the old generator that has been updated t − 1 times. Both generators generate sample data to be fitted to the real data. In order to evaluate the fitting degree of the generated samples to real data, we propose a maximum likelihood estimation and the negative-log-likelihood (NLL) function, as follows:

(10) $$NLL=-\frac{1}{N_{t}}\left[\sum_{i=1}^{N}logG_{\theta }(P_{fate}|P_{i})\right]$$

where $N_{t}$ represents the number of samples arriving at the t-th time, $P_{fate}$ represents the generated data samples by the generator, and $P_{i}$ represents the medical examination data of the i-th patient in the data arriving at the t-th time. If the condition ${NLL}_{new}<{NLL}_{t-1}$ is met, the data will enter the online updating framework of the model; otherwise, the model will directly learn from the new data.

This study designs an experience replay strategy similar to generative adversarial networks. It integrates generated data samples with new visit data to form a fusion dataset. In the generative adversarial networks, the generator attempts to capture the distribution of EHR data from the patients, while the discriminator tries to assess the probability that input samples come from real data rather than the generator.

In order to enable the model to use the features learned from previous tasks to train the new tasks, we propose a feature distillation strategy to extract features generated from previous tasks. Before starting a new task, we freeze the feature extractor and create a new identical feature extractor. After the two feature extractors perform feature extraction separately, the loss function is computed between them to prevent forgetting. The loss function can be calculated as follows:

(11) $$L_{F_{t}}=\frac{1}{N}\sum_{rec\in {REC}_{t}}\left|\right|F_{t}(rec)-F_{t-1}(rec){\left|\right|}_{2}$$

where $F_{t}$ represents the t-th feature extractor, ${REC}_{t}$ represents the collection of multivisit EHR for patients in task t, and rec represents the multivisit EHR of the patient.

In this study, the generator consists of three linear layers, and each is followed by an activation function. It concatenates random noise vectors and is-hyper vectors to form the input vectors, which are then passed through the network composed of linear layers and activation functions to generate normalized output features. The loss function of the generator can be calculated as follows:

(12) $$L_{G_{t}}=-E_{z\sim q_{z},is-hyper\in label}{[D}_{t}(is-hyper,G_{t}\left(is-hyper,z\right))]$$

where $G_{t}$ represents the generator incrementally generated at the t-th time, and $q_{z}$ is a randomly generated noise variable, which is input together with the sample label into the generator to obtain the generated data sample. $D_{t}$ represents the discriminator incrementally generated at the t-th time. The discriminator calculates the probability of the sample coming from real data or the generator by, respectively, receiving the generated data from $G_{t}$ and the visit record data from the patient. It consists of two linear layers, and each is followed by an activation function. It receives real data and generated data, extracts hidden features from the data, and calculates the real probability and class classification probability of the generated data through validity scores. Through adversarial learning, both the generator and discriminator are alternately updated to continuously improve the quality of the generated data. The loss function of the discriminator can be calculated as follows:

(13) $$\begin{array}{c} L_{D_{t}}=E_{z\sim q_{z},is-hyper\in label}{[D}_{t}(is-hyper,G_{t}\left(is-hyper,z\right))]\\ -E{[D}_{t}(is-hyper,F_{t}\left(rec\right))] \end{array}$$

By training the discriminator $G_{t}$ to maximize the probability of discriminating between samples from real data and training samples, we achieve alternating updates of the generator and discriminator in the t-th incremental process in an adversarial learning manner.

In order to avoid model overfitting on new data when using old knowledge to predict new data, we design a replay alignment mechanism. This mechanism generates a replay generator $G_{s}$ with the exact same network structure as the current generator $G_{t}$. Before the start of the t-th incremental task, the noise variables, parameter space, and conditional space of the two generators are aligned.

During the training of the generator, EHR data features are generated by inputting the same hypertension category and noise variables as the generator. The loss function is minimized to align the medical examination record features of the current generator as closely as possible with those of the replay generator, thereby preventing the hypertension monitoring model from overfitting to the training data completely. The loss function of the replay alignment mechanism can be calculated as follows:

(14) $$L_{RA}=E_{z\sim q_{z},is-hyper\in label}{[||G}_{t}\left(is-hyper\right)-G_{s}\left(is-hyper,z\right){\left|\right|}_{2}^{2}]$$

### 3.5. The Pseudo-Code of Model

Algorithm 1 presents the core algorithm for the proposed model. At the beginning of the algorithm, the feature extractor and generator are initialized. As the segmented dataset is continuously fed into the algorithm, the generator, discriminator, and feature extractor are continuously updated. Ultimately, the agent will predict the hypertension risk of the patient and produce the output.
**Algorithm 1:** Online Learning with Generative Feature Replay for Hypertension Risk Prediction. 1:**Input:** Sequence $D_{1},\ldots ..,D_{T}$
 2:**Require:** Feature extractor $F_{0}$, Generator $G_{0}$
 3:**for** t = 1, …, T 4:  **if** t == 1 then 5:    train $F_{1}\leftarrow F_{0}\;with$ $D_{1}$
 6:    train $G_{1}\leftarrow G_{0}\;with\;F_{1}$
 7:  **else** 8:    **If** ${NLL}_{t}\leq {NLL}_{t-1}$ then 9:      $train\;F_{t}\leftarrow F_{t-1}\;\;with\;\;D_{t}$
10:      $train\;G_{t}\leftarrow G_{t-1}\;with\;\;F_{t}$
11:    **else**12:      set $G_{t}=G_{t-1}$
13:  **Repeat**14:    ${state}_{t}^{n}\leftarrow {item\left[i\right]}_{i}^{n}\;and\;P_{i}^{n}$
15:    choose action $a_{t}$ and provide reword16:    $gap\leftarrow record+Q\left(s_{t+1},a_{t+1}\right)-Q(s_{t},a_{t})\;$
17:    put sample into replay buffer18:    $L_{RA}\leftarrow G_{t}-G_{s}$
19:  **end Repeat**20:**end for**

## 4. Experiments

### 4.1. Dataset

This study uses personal physical examination records obtained from the public dataset MIMIC-III as the dataset for our experiments. The MIMIC-III dataset is widely used and contains clinical data from tens of thousands of patients spanning from 2001 to 2012. According to the 2023 edition of the Chinese Guidelines for Prevention and Treatment of Hypertension, hypertension is associated with various risk factors such as “daily blood pressure fluctuations”, “abnormal heart rate”, “obesity”, “irregular lifestyle”, and “age”. Based on these risk factors, 20 attributes were chosen from the MIMIC-III dataset and all attributes are listed in Table 1. The attribute “age” corresponds to the risk factor “age”. The attributes “heart rate”, “respiratory rate”, “heart rhythm”, “low heart rate (LHR)”, “high heart rate (HHR)”, “low respiratory rate (LRR)”, “high respiratory rate (HRR)”, and “respiratory pattern” correspond to the risk factor “abnormal heart rate”. The attributes “low blood oxygen saturation (LBOS)”, “high blood oxygen saturation (HBOS)”, “systolic blood pressure (SBP)”, “diastolic blood pressure (DBP)”, “mean arterial blood pressure (MBP)”, “central venous blood pressure (CVBP)”, “low arterial blood pressure (LABP)”, and “high arterial blood pressure (HABP)” correspond to the risk factor “daily blood pressure fluctuations”. The attribute “life pattern” corresponds to the risk factor “irregular lifestyle” and the attribute “weight” corresponds to the risk factor “obesity”. In addition, these attributes and “temperature” reflect the basic lifestyle and physical state of the patient.

This study uses a multivisit dataset based on the number of personal physical examination records. Table 2 shows the statistics of the multivisit samples; as the number of visits increases, the number of patients decreases. Clearly, the availability of multivisit samples for patients each year remains scarce.

**Figure 3:**
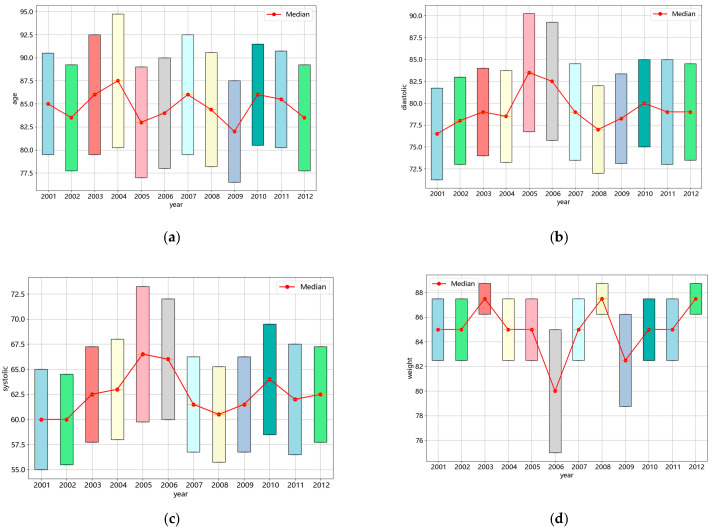
Distribution of characteristics over 12 years. (**a**) Distribution of age over 12 years; (**b**) distribution of diastolic blood pressure over 12 years; (**c**) distribution of systolic blood pressure over 12 years; (**d**) distribution of weight over 12 years.

In order to simulate a few-shot, incremental, and nonstationary EHR generation scenario, this study divides the MIMIC-III dataset into 12 batches of continuous dataset based on the year of data generation. Table 3 shows the number of patients and the number of records for each of the 12 years. It can be seen that the number of EHRs available per year does not exceed 610, and records of multivisit are scarce. Taking age, diastolic blood pressure, systolic blood pressure, and weight as examples, the distribution of the characteristics of patients examined in each of the 12 years is shown in Figure 3. It can be observed that regardless of the examination value, there is a consistent range of distribution. However, the examination values of most patients still undergo changes, which are irregular and present in each batch of data. Therefore, a fixed hypertension prediction model is insufficient to meet the needs of patients in real-world scenarios. And an adaptive updating model that can adapt to continuous data streams is still of significant research value.

### 4.2. Baselines

In this study, the proposed model HRP−OG is compared with the following baselines. All methods are implemented in Pytorch and trained on an NVIDIA GeForce RTX 3070Ti. Their source codes are available in original papers:

(1) LSTM [40] is a predictive model based on sequential data that can handle irregular time intervals in patient examination records. In the experiment, the learning rate is set to 1 × 10^−3^, the hidden layer size is set to 100, and the number of hidden layers is set to 2.
(2) KNN-SVM [41] is a model that utilizes grid search technique for hyperparameter tuning and can handle imbalanced data for prediction. In the experiment, the regularization parameter is set to 1, and the kernel function is set to radial basis function.
(3) ConCare [42] is a multichannel longitudinal disease risk prediction model. It adopts improved multihead self-attention to extract the interdependencies among time series of dynamic features as well as static baseline information for disease prediction. In the experiment, the learning rate was set to 1 × 10^−3^, the input feature dimension was 76, and the number of heads in the multihead attention mechanism was set to 4.
(4) LightGBM [43] is a gradient boosting framework that builds multiple decision trees and learns from the gradients to handle few-shot learning. In the experiment, the learning rate is set to 0.1, the boosting type is set to gradient boosting decision tree, the number of trees built is set to 10, and the maximum number of leaf nodes is set to 31.
(5) KNN-LightGBM [44] is a disease risk prediction model based on deep learning. It predicts hypertension using a hybrid deep neural network combining KNN and LightGBM based on single-instance EHR. In this experiment, the number of nearest neighbors is set to 5, the learning rate is set to 1 × 10^−3^, and the distance metric used Minkowski.
(6) SACNN-SOA [45] is a chronic disease diagnosis based on the Self-Attention Convolutional Neural Network optimized with Season Optimization Algorithm, which uses IoT and cloud computing in the Smart Medical Big Data health care system. In this experiment, the learning rate is set to 1 × 10^−5^, batch size is set to 20, and the number of convolutional layers is 9.

### 4.3. Metrics

This paper uses the accuracy, precision recall AUC (PR-AUC), and ROC-AUC to measure experimental results.

Accuracy quantifies the proportion of correct predictions in the overall test set, which can demonstrate the reliability of the model’s prediction results. It can be calculated as follows:

(15) $$Accuracy=\frac{TN+TP}{TN+TP+FN+FP}$$

where TP represents the true positive, FP represents the false positive, TN represents true negative, and FN represents the false negative.

Precision, recall, and FPR can be calculated as follows:

(16) $$Precision=\frac{TP}{TP+FP},\;Recall=\frac{TP}{TP+FN},\;FPR=\frac{FP}{TN+FP}$$

PR-AUC is the area under the precision–recall curve, which can provide a comprehensive model evaluation that combines precision rate and recall rate. In the case of imbalanced data, PR-AUC can effectively reflect the performance of the model. It can be calculated as follows:

(17) $$PR-AUC=\int_{0}^{1}Precision(Recall)dRecall$$

ROC-AUC is the area under the ROC (receiver operating characteristic) curve, which can also provide a comprehensive model evaluation. Unlike PR-AUC, which focuses on the accuracy of the model in predicting positive samples, ROC-AUC is suitable for scenarios where the model is expected to perform well on both positive and negative samples (e.g., reducing the misdiagnosis rate). Therefore, it can provide a relatively robust evaluation for imbalanced datasets. ROC-AUC can be calculated as follows:

(18) $$ROC-AUC=\int_{0}^{1}Recall(FPR)dFPR$$

### 4.4. Results

In the experiments, the multivisit records are divided into 12 batches by year to simulate the scenario of incremental data in a nonstationary environment. Each batch of data is randomly split into training set, test set, and validation set in a ratio of 3:1:1. The results of 5-fold cross-validation are shown in Table 4 and Table 5, which present the accuracy and PR-AUC values of different models, respectively. We recorded the performance changes of the models over 12 stages and calculated the average performance over these 12 stages. Despite fluctuations in the data, all models showed performance variations within a certain range, indicating differences in their ability to learn hypertension features.

In all the experiments, SACNN-SOA showed the worst performance; both the accuracy value and the PR-AUC value were the lowest among all the models. The reason for this is related to the characteristics of the SACNN-SOA model; as described in its paper, the model is based on big data and cloud computing platforms, and requires a large number of data samples for training. However, due to the limited number of samples in our experiments, this model could not accurately capture the feature distribution of hypertensive patients.

In contrast, LightGBM performed excellently in several comparative experiments, achieving accuracy and PR-AUC values of 0.6086 and 0.8043, respectively, indicating its ability to effectively learn hypertension features from multivisit records.

The KNN-LightGBM hybrid prediction model, which combines LightGBM and KNN, is expected to perform better than LightGBM alone according to its paper. However, in our experiment, KNN-LightGBM and LightGBM have similar predictive performance. Comparing the accuracy and PR-AUC indicators across several experiments, it can be seen that KNN-LightGBM achieved better performance in the seventh, eighth, and tenth stages, while LightGBM performed better in the second, fourth, and sixth stages. Overall, the accuracy and PR-AUC of LightGBM were slightly higher than those of KNN-LightGBM. The reason why each of the two models can outperform the other in certain stages is that KNN and LightGBM are trained simultaneously, and their final results are weighted averages. In some data streams, the weights of the two models cannot be effectively allocated, preventing the model from achieving better performance.

KNN-SVM is also a predictive model that combines KNN. In our experiment, the overall performance of the model is slightly better than KNN-LightGBM, and the model achieves better predictive results in the first, ninth, eleventh, and twelfth stages. The reason is that this model uses a more complex ensemble learning method, which can better integrate multiple modules.

LSTM does not perform well on either indicator, possibly because the model is relatively simple and cannot effectively capture long sequence features from few-shot EHR samples.

The ConCare model performs well on the PR-AUC indicator but poorly on the accuracy indicator, suggesting that while the model does not predict overall physical examination data well, it is better at identifying hypertensive patients, failing to effectively recognize nonhypertensive samples.

Finally, the model we proposed achieves the best results across continuous testing of 12 data batches. In terms of the accuracy indicator, it outperformed other models by more than 20% at each stage. In terms of the PR-AUC indicator, it outperformed other models by more than 10% at each stage, and the average performance on both indicators is far superior to that of other models, demonstrating the effectiveness in learning data features from a few-shot and nonstationary environment.

## 5. Discussion

This study conducts ablation experiments on our model HRM−AR, and three HRM−AR variants models are designed as follows:

1. ${HRP-OG}_{retrain}$. Based on HRP−OG, this variant directly abandons the knowledge learned from historical data, instead learning from new data and making predictions. This variant is used to justify the need for model updates.
2. ${HRP-OG}_{G-}.$ Based on HRP−OG, the online updating module is removed. The reinforcement learning module is directly used for training and testing. This variant is used to demonstrate the necessity and effectiveness of the generative feature replay.
3. ${HRP-OG}_{N-}.$ Based on HRP−OG, the data evaluation module is removed. The reinforcement learning module and generative feature replay module are directly used for training and testing. This variant is used to demonstrate the necessity of the sample evaluation module.

**Figure 4:**
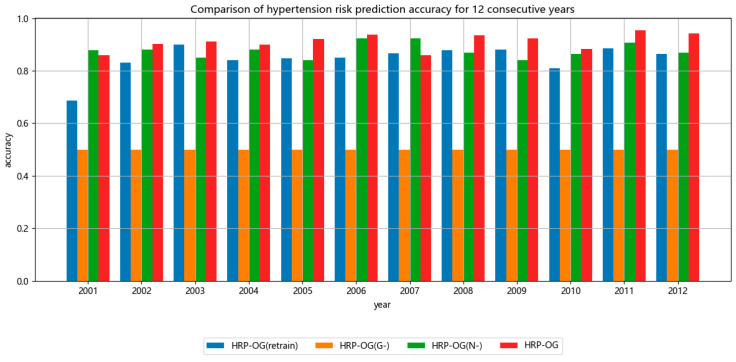
Accuracy value of ablation experiments.

**Figure 5:**
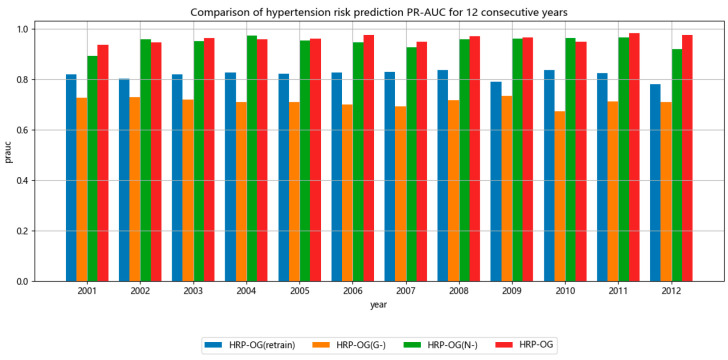
PR-AUC value of ablation experiments.

Figure 4 and Figure 5 show the results of the ablation study, displaying the performance of several models evaluated based on the accuracy and PR-AUC metrics, respectively. The performance of the models demonstrated by both metrics is similar. The result of ${HRP-OG}_{G-}$ is the worst, even worse than most of the instance-based baseline methods shown in Table 4 and Table 5. This indicates that using past models directly cannot make good predictions on new data, as the old models cannot effectively capture the features of hypertension patients on few-shot datasets. The result of ${HRP-OG}_{retrain}$ is close to most of the instance-based baseline methods shown in Table 4 and Table 5, indicating that the ability of the model to capture features on few-shot datasets is relatively close. They can predict the hypertension risk, but the accuracy of the predictions could not be compared to the models incorporating historical visit records. This underscores the necessity of online updates to the model in response to changes in the data. The result of ${HRP-OG}_{N-}$ are comparable to HRP−OG, and even exceed it in some stages. This indicates that the model can effectively extract feature distributions from the data. However, in few-shot datasets, there are biases in the data features. Thus, continuous effective model updates are impossible without assessing data quality. This highlights the necessity of sample selection based on maximum likelihood estimation.

Overall, in terms of the accuracy indicator, HRP−OG improves by 7% compared to ${HRP-OG}_{retrain}$, by 41% compared to ${HRP-OG}_{G-}$, and by 4% compared to ${HRP-OG}_{N-}$. In terms of the PR-AUC indicator, HRP−OG improves by 14% compared to ${HRP-OG}_{retrain}$, by 25% compared to ${HRP-OG}_{G-}$, and by 2% compared to ${HRP-OG}_{N-}$.

**Figure 6:**
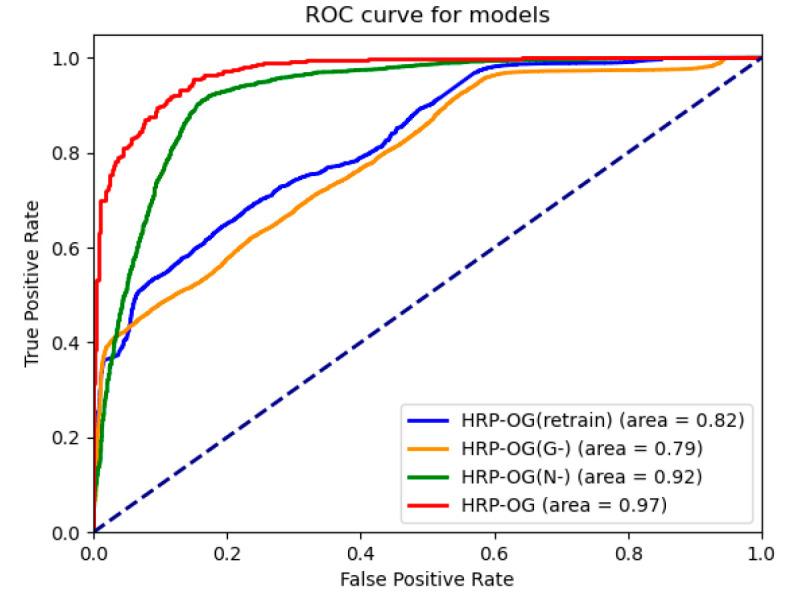
ROC curve of ablation experiments.

Figure 6 shows the ROC curves of the models, illustrating the overall ROC curves of the HRP−OG model and its variants across 12 batches of data. The value for HRP−OG is the highest, reaching 0.97, indicating that our model can effectively capture the characteristics of hypertensive patients in a few-shot and nonstationary environment, achieving accurate predictions for hypertensive patients. The ROC value for ${HRP-OG}_{N-}$ is 0.92. Although it is not as high as HRP−OG, it still demonstrates effective prediction capabilities. However, due to the lack of evaluation for new data, its overall performance is weaker. The ROC value for ${HRP-OG}_{retrain}$ is only 0.82, indicating that it can achieve limited prediction effectiveness, but its accuracy is still constrained by the few-shot size. The ROC value for ${HRP-OG}_{G-}$ is the lowest, at only 0.79, which is even lower than that of the retrained model. This also indicates that directly using an old model to train new data is not feasible, as it can lead to overfitting under few-shot conditions and affect prediction accuracy.

## 6. Conclusions

This study designed and implemented an online hypertension risk monitoring model, HRP-OG, based on few-shot multivisit data. Based on a reinforcement learning framework, we constructed the hypertension risk prediction model, enabling it to extract hypertension features and perform risk prediction. A generative feature replay module was designed to apply knowledge learned from historical data to new data domains, facilitating model updates. Furthermore, a data quality assessment method was proposed to avoid bias due to large gaps between the feature distribution of historical and new data, further enhancing the effectiveness of model updates. Experimental results on the MIMIC-III dataset validated our model, and the average accuracy of each stage reached 0.96, which can effectively identify hypertensive patients. In the future, we plan to incorporate a wider range of diseases into our model to improve its practicality. Meanwhile, more advanced deep learning methods [46] will be considered to further improve the performance of the model.

## Figures and Tables

**Table 1:** Statistical information of hypertension dataset.

Features	Is-Hyper = 0	Is-Hyper = 1
mean # of age	67.9	70.1
mean # of heart rate	88.9	84.7
mean # of respiratory rate	19.0	18.9
mean # of heart rhythm	None	None
mean # of LHR	54.6	54.4
mean # of HHR	121.8	121.4
mean # of LBOS	89.7	89.7
mean # of HBOS	99.0	99.2
mean # of LRR	9.2	9.3
mean # of HRR	33.4	33.0
mean # of SBP	118.2	123.4
mean # of DBP	62.2	64.6
mean # of MBP	79.1	82.3
mean # of life pattern	None	None
mean # of weight	93.1	95.5
mean # of CVBP	10.9	11.0
mean # of LABP	83.1	83.3
mean # of HABP	147.5	149.6
mean # of breathing pattern	None	None
mean # of temperature	37.9	36.0

**Table 2:** Statistics of the multivisit samples.

Number of Visits	Number of Patients	Number of Records
2	4479	8958
3	1124	3372
4–6	672	2915
6–8	137	871
8–10	47	397
10–15	41	457
>15	15	350
all	6515	17,320

**Table 3:** Distribution of the multivisit samples over 12 years.

Year	Number of Patients	Number of Records
2001	538	1247
2002	610	1585
2003	601	1639
2004	612	1638
2005	607	1652
2006	576	1551
2007	562	1493
2008	569	1509
2009	600	1596
2010	588	1518
2011	572	1470
2012	79	421

**Table 4:** Accuracy value of comparison experiments.

Method	LSTM	KNN-SVM	ConCare	LightGBM	KNN-LightGBM	SACNN-SOA	HRP-OG
1	0.5286	0.5938	0.5938	0.5781	0.5625	0.5000	0.8594
2	0.4818	0.5000	0.4922	0.5990	0.5938	0.4844	0.9010
3	0.5353	0.5588	0.6059	0.5588	0.5706	0.4971	0.9118
4	0.5673	0.5577	0.5577	0.5817	0.5625	0.4976	0.8990
5	0.6500	0.7043	0.7043	0.6783	0.6217	0.5000	0.9196
6	0.5807	0.6366	0.6211	0.7267	0.6708	0.4783	0.9379
7	0.5119	0.4908	0.4881	0.5381	0.5857	0.5000	0.8595
8	0.5601	0.5902	0.5847	0.5738	0.6066	0.4918	0.9344
9	0.6083	0.6178	0.4873	0.5414	0.5350	0.4873	0.9236
10	0.5497	0.5789	0.5819	0.5614	0.6082	0.4912	0.8830
11	0.6824	0.7044	0.6761	0.6855	0.6352	0.4937	0.9528
12	0.6675	0.6806	0.6780	0.6806	0.6073	0.4843	0.9424
average	0.5770	0.6012	0.5893	0.6086	0.5967	0.4921	0.9104

**Table 5:** PR-AUC value of comparison experiments.

Method	LSTM	KNN-SVM	ConCare	LightGBM	KNN-LightGBM	SACNN-SOA	HRP-OG
1	0.7917	0.7970	0.7969	0.7891	0.7813	0.7057	0.9349
2	0.7370	0.7500	0.7448	0.7995	0.7969	0.7448	0.9453
3	0.8029	0.7765	0.8029	0.7794	0.7853	0.7176	0.9618
4	0.7813	0.7787	0.7788	0.7909	0.7813	0.7308	0.9567
5	0.8532	0.8521	0.8522	0.8391	0.8109	0.6630	0.9609
6	0.8106	0.8168	0.8106	0.8634	0.8354	0.6988	0.9752
7	0.7452	0.7453	0.7452	0.7690	0.7929	0.7476	0.9476
8	0.7951	0.7950	0.7951	0.7868	0.8033	0.7077	0.9699
9	0.8089	0.8089	0.8089	0.7707	0.7675	0.6975	0.9650
10	0.7895	0.7996	0.7895	0.7807	0.8041	0.7018	0.9474
11	0.8491	0.8522	0.8491	0.8427	0.8176	0.6730	0.9811
12	0.8403	0.8413	0.8403	0.8403	0.8036	0.6990	0.9738
average	0.8003	0.8011	0.8012	0.8043	0.7983	0.7073	0.9610

## Data Availability

Dataset available on request from the corresponding authors J.C. (chenjianhui@bjut.edu.cn) and H.Y. (yhk-yhk@emails.bjut.edu.cn).
